# Glycosaminoglycans Targeted by Colchicine in MCF-7 Cells

**DOI:** 10.3390/pharmaceutics17111368

**Published:** 2025-10-23

**Authors:** Magdalena Czarnecka-Czapczyńska, Agnieszka Przygórzewska, Klaudia Dynarowicz, Dorota Bartusik-Aebisher, David Aebisher, Aleksandra Kawczyk-Krupka

**Affiliations:** 1 Department of Internal Diseases, Angiology and Physical Medicine, Center for Laser Diagnostics and Therapy, Medical University of Silesia, Batorego 15, 41-902 Bytom, Poland; magdalena.czarnecka921114@gmail.com (M.C.-C.); akawczyk@gmail.com (A.K.-K.); 2 English Division Students Science Club, Faculty of Medicine, Collegium Medicum, University of Rzeszów, 35-310 Rzeszów, Poland; 3 Department of Biochemistry and General Chemistry, Faculty of Medicine, Collegium Medicum, University of Rzeszów, 35-310 Rzeszów, Poland; kdynarowicz@ur.edu.pl (K.D.);; 4 Department of Photomedicine and Physical Chemistry, Faculty of Medicine, Collegium Medicum, University of Rzeszów, 35-310 Rzeszów, Poland

**Keywords:** colchicine, glycosaminoglycan, breast cancer cells MCF-7, magnetic resonance imaging, fixed charge density

## Abstract

**Background**: Breast cancer is the most common cancer diagnosis and the second leading cause of cancer-related death in women. Breast cancer is a major health burden worldwide. Advances in breast cancer detection and treatment have contributed to improving the rate of survival, although mortality rates remain significantly high. Despite all these advances, more efficient diagnostic methods and effective treatments are necessary. Colchicine is a natural alkaloid with strong antimitotic activity, but its potential effects on extracellular matrix components in cancer remain poorly understood. **Objective**: This study aimed to investigate the influence of colchicine on glycosaminoglycan (*GAG*) concentrations and cell viability in MCF-7 breast cancer cells cultured in a three-dimensional (3D) hollow fiber bioreactor system. **Methods**: Magnetic resonance imaging (MRI) was applied as a non-invasive technique to quantify *GAG* levels through fixed charge density (*FCD*) and *T*_1_ relaxation mapping. MCF-7 HER-2-overexpressing and HER-2-negative cells were treated with 1000 nM colchicine for 72 h, and cell viability was assessed in parallel with *GAG* measurements. **Results**: Colchicine significantly reduced cell viability and altered *GAG* concentrations. HER-2-overexpressing MCF-7 cells exhibited higher baseline *GAG* levels than HER-2-negative controls, and colchicine decreased the *GAG* content in both lines. **Conclusions**: Colchicine reduces viability and modifies *GAG* concentrations in 3D cultures of MCF-7 cells. The use of MRI provides a reproducible, non-destructive tool for monitoring extracellular matrix changes, offering a novel methodological approach for studying drug effects in physiologically relevant cancer models.

## 1. Introduction

Numerous molecular markers exhibit varying capacities in detecting breast cancer However, none of the current markers individually are able to provide a precise diagnosis and discriminate accurately between indolent and aggressive cancers. Simultaneous detection of multiple indicators of breast cancer’s presence and progression are needed to provide more efficient early-stage diagnosis, predict the disease course, and develop more effective therapy. The purpose of this project is to develop a molecular imaging and spectroscopy technique which is able to detect two or more molecular markers simultaneously. The proposed work in the project will provide a long-term strategy to study molecular imaging and create functionalized nanoparticles targeted at antigens expressed by breast cancer.

Colchicine is a tropolone alkaloid extracted mainly from *Colchicum autumnale*, known for centuries and traditionally used to treat acute inflammation [1,2,3,4]. It is a water-soluble plant alkaloid with a characteristic structure and biosynthetic pathway [5,6,7,8]. Despite its long history of clinical use, colchicine was only approved by the FDA in 2009 for the treatment of gout, familial Mediterranean fever, and Behçet’s disease [9,10,11]. Recently, increasing interest has been directed toward its potential as an anticancer agent due to its strong antimitotic activity, cytotoxic properties, and ability to induce apoptosis in different tumor cell lines [12,13,14]. The main mechanism of colchicine action involves binding to tubulin and disrupting microtubule polymerization, which, in turn, impairs mitosis and multiple cellular processes such as migration and intracellular transport [15,16,17,18,19,20,21]. By additionally affecting the mitochondrial metabolism and apoptosis pathways, colchicine shows promise as an experimental anticancer drug [22,23,24,25,26,27].

Biopsy is one of the main methods used to diagnose breast cancer. Unfortunately, even when taking many samples, biopsies can still sometimes miss detecting cancer if none of the biopsy needles pass through the affected tissue. In addition to the inaccuracy of the biopsy, it is also highly invasive and uncomfortable for the patient. Some biomarkers such as estrogen receptor alpha (ERa), progesterone receptor (PR), and cyclin D1 show similar patterns of expression in epithelial cells lining breast cysts as malignant epithelial cells in local and invasive ductal breast cancer. Specimens are analyzed by standard immunohistochemistry for ERa, PR, cyclin D1, bcl-2, p53, and ErbB2 expression. ErbB-2 is not expressed in normal control specimens. ErbB-2 is expressed in the same specimens in an increasing proportion of normal breast acini, microcysts, and cancer cells in 36% of specimens with breast cancer.

The emerging field of molecular imaging uses targeted and imaging agents to exploit specific molecular targets, pathways, or cellular processes to generate the images. Molecular imaging promises to play a decisive role in the non-invasive detection of breast cancer since the pathways are relatively well-studied and, consequently, the molecular targets of interest are well known. Numerous molecular markers have been found in human serum, histological specimens that exhibit varying capacities to detect breast cancer. Molecular imaging of the markers promises to improve the quality of patient care dramatically by making it possible to detect tumors much earlier when they are easier to treat and by permitting more precise therapy or surgery. Some researchers have begun investigating such methods for many diseases.

Studies on anticancer activity require experimental models that adequately reflect the tumor microenvironment. Conventional two-dimensional (2D) cultures, although easy to handle, do not reproduce the complex architecture, gradients of oxygen and nutrients, and cellular heterogeneity of tumors in vivo [28,29,30,31,32,33,34]. In contrast, three-dimensional (3D) culture models allow cells to self-organize into spheroids or tissue-like structures, mimicking conditions of hypoxia, necrosis, and an altered drug response [35,36,37,38,39,40,41,42,43,44,45,46]. Among the available technologies, hollow fiber bioreactors (HFB) are particularly advantageous, as they provide high cell density and long-term culture under controlled conditions, enabling the monitoring of dynamic changes in cancer cells [47,48,49,50,51,52,53,54,55,56,57].

**Figure 1:**
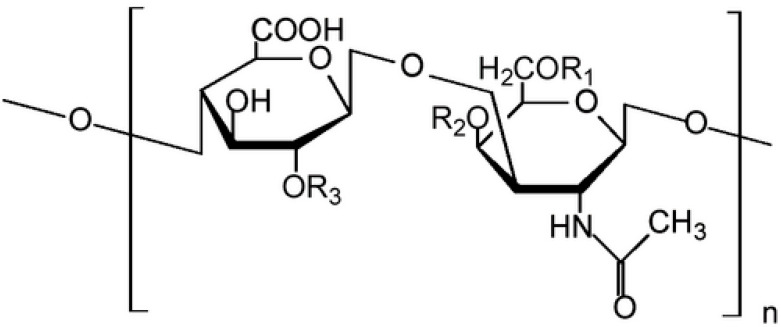
The repeating disaccharide unit (GlcUA(1β→3)GalNAc(1β→4))n of *GAG* sulfate composed of a chain of alternating sugars (N-acetylgalactosamine and glucuronic acid). It is usually found attached to proteins as part of a proteoglycan.

A key aspect of tumor biology is the extracellular matrix, where glycosaminoglycans (*GAG*s) play a central role. These complex polysaccharides, built from repeating disaccharide units (Figure 1), are highly negatively charged and interact with growth factors, cytokines, and cell receptors [58,59,60,61,62,63,64]. *GAG*s regulate numerous physiological and pathological processes, including inflammation, fibrosis, angiogenesis, and cancer progression [65,66,67,68]. During tumor development, their composition and structure undergo significant changes in terms of molecular weight and sulfation patterns [69].

Magnetic resonance imaging (MRI) offers an opportunity to monitor these changes in a non-invasive way. More advanced MRI techniques offer a quantitative assessment of the biochemical composition of cells. This technique allows reproducible and precise measurements of the same sample without causing damage, providing accurate data on *GAG* content through *T*_1_ relaxation mapping and *FCD* calculations. *GAG*s are critical constituents of cells. *GAG* is the oligosaccharide chains of heparan sulfate proteoglycans. The sulfation of HS glycosaminoglycan residues is required for its interaction with various heparin-binding growth factors to promote their biological activities to activate their high-affinity receptor tyrosine kinases.

The effect of *GAG* sulfate in people with osteoarthritis is likely the result of a number of reactions including its anti-inflammatory activity, the stimulation of the synthesis of proteoglycans and hyaluronic acid. First, the amount of collagen increases in all abnormal breast pathologies. This is consistent with known breast pathology, as lesion formation is often accompanied by fibrosis. Fibrosis is a scarring process characterized by an increased stromal component, and thus an accumulation of collagen and *GAG*. The relative increase in collagen is most pronounced in fibrocystic change, a benign condition that can manifest as fibrosis, adenosis (increase in the number of ductules), and/or cyst formation. Fibroadenoma is a benign tumor characterized by both fibroblast and ductal proliferation. Furthermore, these differences have important implications not only for probing and analyzing the developing process of the breast lesion at the molecular level but also for evaluating the histological types and grades of breast diseases.

Since *GAG* have negatively charged side-groups, mobile ions will distribute in the tissue to reflect the local *GAG* concentrations. To obtain this effect, we used charged contrast agent *Gd*(*DTPA*)^2−^. *T*_1_-calculated images can be used to quantify *Gd*(*DTPA*)^2−^, which can be used to quantify *GAG* using a modified electrochemical equilibrium theory. This approach provides high reproducibility, especially in controlled 3D culture systems [70,71]. These measures have great potential to be used as biomarkers to track cellular changes.

**Figure 2:**
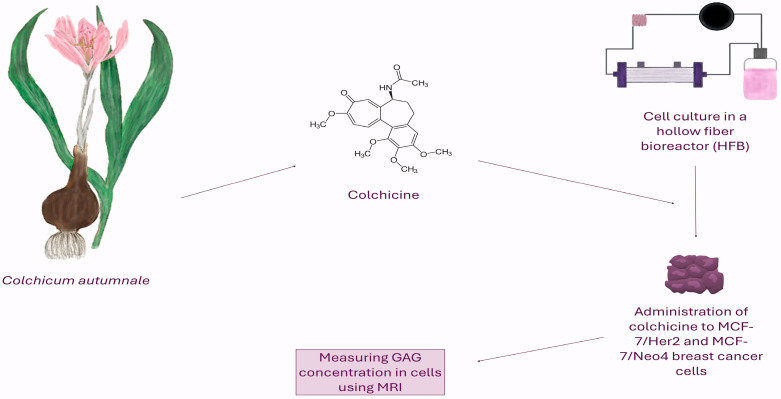
The figure shows a schematic diagram of the experiment. Colchicine, an alkaloid derived from *Colchicum autumnale*, was applied to HMEC, MCF-7/Her2, and MCF-7/Neo4 breast cancer cell cultures conducted in a capillary fiber bioreactor (HFB) system. The aim of the study was to non-invasively monitor changes in glycosaminoglycan (*GAG*) concentration in three-dimensional cell cultures using magnetic resonance imaging (MRI).

The aim of this study was to investigate the effect of colchicine on the viability of both MCF-7(NEO-4) and MCF-7(HER-2) breast cancer cells lines and HMCE breast cells cultured in a 3D hollow fiber bioreactor and to assess associated changes in the *GAG* concentration using MRI (Figure 2). These novel techniques provide numeric measures that correlate with various physiological properties of cells.

## 2. Materials and Methods

### 2.1. Chemicals and Aparature

All used chemicals were of analytical grade. Colchicine was purchased from Sigma-Aldrich (Warsaw, Poland). Dulbecco’s Modified Eagle’s Medium (DMEM), fetal bovine serum (FBS), trypsin, and ethylenediaminetetraacetic acid (EDTA) were purchased from Sigma Aldrich (Warsaw, Poland). HMEC and breast cancer cells were obtained from American Type Cell Culture (ATCC, Manassas, VA, USA). Phosphate-buffered saline without calcium and magnesium ions, L-glutamine, and penicillin were obtained from Sigma Aldrich (Warsaw, Poland).

### 2.2. Cell Culture Methods

MCF-7/Her2 cells were cultured in RPMI 1640 medium (Biofluids) containing 5% fetal calf serum (FBS), 2 mM L-glutamine, 50 units/mL penicillin, and 50 µg/mL streptomycin. MCF-7/Neo4 cells were maintained in DMEM with elevated glucose (4500 mg/L), enriched with 4-fold concentrations of amino acids and vitamins, 2 mM L-glutamine, 50 µg/mL streptomycin sulfate, 10 µg/mL gentamicin sulfate, and 1.5 IU/mL in-terleukin-2 (IL-2). For Human Mammary Epithelial Cells, we used Mammary Epithelial Cell complete medium (basal medium plus growth kit) as an optimal serum-free culture model.

Cells were cultured in a hollow fiber bioreactor system (HFBR, FiberCell Systems Inc., New Market, MD, USA) consisting of a polysulfone tube (inner diameter 10 mm, length 160 mm) containing a porous capillary fiber (inner diameter 700 µm, outer diameter 1300 µm, and pore size 0.1 µm), sealed at both ends with silicone rubber cement and connected to a perfusion circuit.

Prior to cell inoculation, the system was prepared by a three-step pre-culture procedure aimed at (1) removing the wetting agent from the fibers, (2) stabilizing the system with growth medium and serum proteins, and (3) confirming the sterility and tightness of the system. The fibers were incubated successively in 100–250 mL of phosphate-buffered saline (PBS), then in serum-free medium, and finally in complete culture medium. Each step lasted at least 24 h. After the bioreactor was activated, the cells (30 × 10^6^) were resuspended in the medium and injected into the extracapillary space of the cartridge.

Two hours after inoculation, circulation of the medium was started using a peristaltic pump (Fisher Scientific, Hampton, NH, USA), located in an incubator maintaining a 5% CO_2_/95% air and 37 °C temperature. The medium was partially recycled, while an additional 45 mL of fresh medium was supplied to the system. Ho-dowel of cells was carried out for up to 4 weeks, with sterile conditions and constant physicochemical parameters, including pH maintained in the range of 6.8–7.0.

### 2.3. Cells Treatment

The cells were treated 6 weeks after the cells’ inoculation in the HFB. The growth of MCF-7 cells was inhibited using 1000 nM concentrations of colchicine (colchicine was purchased from Sigma Aldrich, Saint Louis, MO, USA).

### 2.4. Methods for Counting Cells in a Bioreactor

Two different methods were used to assess cell counts:

Method I: Trypan blue staining followed by visual counting using a hemocytometer (Hausser Scientific, Horsham, PA, USA) was performed daily until cell density reached 10^7^ cells/mL. A total of 50 μL of cell suspension and 50 mL of trypan blue solution were placed in 2900 μL of suspension buffer. The sample was mixed and left for 5 min, then placed on a hemocytometer, and the cells were counted. Method II: We also used glucose monitoring to count cells, assuming that 1 g of glucose consumption per day corresponds to 109 cells inside the bioreactor.

### 2.5. Magnetic Resonance Imaging

During MRI experiments, hollow fiber bioreactors were maintained under incubator-like conditions (37 °C, 5% CO_2_, and 95% air). All MRI experiments were performed using 1.5 T. The post-longitudinal relaxation time of water protons (*T*_1_) of both cell lines was measured using an Inversion Recovery (IR) pulse sequence with an echo time (TE) of 16.5 ms, a repetition time (TR) of 8000 ms, and 8 inversion times (TI): 10, 100, 200, 400, 800, 1000, 2000, and 4000 ms. *T*_2_ relaxation times were measured using a multi-echo spin–echo (SE) pulse sequence with a TR of 8000 ms and 11 echoes 10 ms apart with a first TE of 16.5 ms. The imaging plane was perpendicular to the long axis of the bioreactor. The field of view was 3 × 3 cm, the cross-sectional thickness was 2 mm, and the matrix size was 256 × 256. Ten post-echo images were taken in each series of measurements. Each measurement was performed three times.

### 2.6. Measurement of Glycosaminoglycans

*GAG* concentration was calculated based on the value of the charge density constant (*FCD*), which was measured by washing the culture with *Gd*(*DTPA*)^2−^ (Berlex, Montville, NJ, USA). Measured *T*_1_ was used to calculate *Gd*(*DTPA*)^2−^ and *GAG*, as previously described [71].

*FCD* can be expressed as

(1) $$FCD_{tissue}=-2{\left[Na^{+}\right]}_{bath}\left(\sqrt{\frac{{\left[Gd{\left(DTPA\right)}^{2-}\right]}_{tissue}}{{\left[Gd{\left(DTPA\right)}^{2-}\right]}_{bath}}}-\sqrt{\frac{{\left[Gd{\left(DTPA\right)}^{2-}\right]}_{bath}}{{\left[Gd{\left(DTPA\right)}^{2-}\right]}_{tissue}}}\right)$$

where

$${\left[Gd{\left(GDPA\right)}^{2-}\right]}_{tissue}=\frac{1}{R}\left(\frac{1}{(post\;Gd)T_{1(tissue)}}-\frac{1}{(pre\;Gd)T_{1\left(tissue\right)}}\right)$$

and

$${\left[Gd{\left(DTPA\right)}^{2-}\right]}_{bath}=\frac{1}{R}\left(\frac{1}{(post\;Gd)T_{1(bath)}}-\frac{1}{(pre\;Gd)T_{1\left(bath\right)}}\right)$$

where

*bath*—medium around the breast cancer cells;
*R*—relaxivity (mmol/L/s);
*tissue*—breast cancer cell tissues;
${\left[Na^{+}\right]}_{bath}$—concentration of Na+ ions in *bath*, 154 (mmol/L);
$(post\;Gd)T_{1(tissue)}$—*T*_1_ relaxation time of the breast cancer cells after administration of *Gd*(*DTPA*)^2−^ solution in sec;
$T_{1(tissue)}$—*T*_1_ relaxation time of the breast cancer cells before administration of *Gd*(*DTPA*)^2−^ solution in sec;
$(post\;Gd)T_{1(bath)}$—*T*_1_ relaxation time of the *bath* after administration of *Gd*(*DTPA*)^2−^ solution in sec;
$T_{1(bath)}$—*T*_1_ relaxation time of *bath* before administration of *Gd*(*DTPA*)^2−^ solution in sec.

The calculated *FCD* was converted to *GAG* concentration, according to Equation (2):

(2) $$GAG=FCD\left(\frac{502.5}{-2}\right)$$

where

GAG—Glycosaminoglycan concentration (mg/L);
FCD—Fixed charge density (mmol/L);
502.5—Molecular weight of *GAG* in (mg/mmol).

To measure *GAG* concentration, *Gd*(*DTPA*)^2−^ solution was injected into the bioreactor perfusion tubing flow of the medium. The injected volume was calculated to give the final concentration of 2 mmol/L *Gd*(*DTPA*)^2−^.

### 2.7. Statistical Analysis

All statistical analyses were performed using Statistica 13.3. Values were expressed as mean ± SD.

## 3. Results

*T*_1_-weighted MR images were used to allow the computation of the *GAG* concentration in breast cancer cells. The total cell content increased between weeks 1 and 6 from 0.5 × 10^7^ to 1 × 10^9^. The data show that *GAG* content can be calculated in vitro in healthy and cancerous 3D cell cultures treated with colchicine and with the use of *Gd*(*DTPA*)^2−^. The data also suggest that *GAG* distribution can be nonhomogeneous. After equilibration with *Gd*(*DTPA*)^2−^, all values of *T*_1_ were dominated by *Gd*(*DTPA*)^2−^ and became shorter While it is not yet possible to quantify the absolute *GAG* concentration in vivo, these data are encouraging, as we have an opportunity to visualize the relative *GAG* distribution in vitro.

We cultured human 3D breast cancer cells using a fiber bioreactor for six weeks. The concentration of cells in the bioreactor was sufficient for an MRI. Multiple MRI measurements of the same cells were performed. MRI tissue parameters of HMEC, MCF-7 HER-2, and MCF-7 Neo4 breast cell lines are presented in Table 1 and Table 2, respectively. The viability of cells before treatment was high (Table 1). The growth of MCF-7 cells was inhibited for 72 h using 1000 nM concentrations of the water solution of colchicine and decreased from 1 × 10^9^ to 1 × 10^8^ cels/mL. The viability of cells after treatment showed decreased values (Table 2). We measured the *T*_1_ MRI of HMEC, MCF-7 HER-2-positive, and MCF-7 (Neo-4) HER-2-negative breast cancer cell lines in both untreated and colchicine-treated cells after 1, 3, and 6 weeks. The value of *T*_1_ decreased faster between 1 and 3 weeks than at week 6. The cell culture at 1–3 weeks was less dense than after 6 weeks (Table 3). *T*_2_ MRI tissue parameters of HMEC, MCF-7 HER-2-positive, and MCF-7 (Neo-4) HER-2-negative breast cancer cell lines for both untreated and colchicine-treated cells are presented in Table 4. At week 6, the value of *T*_2_ is decreased.

Therefore, we perform the calculation of *GAG* for cells with a significant decrease of *T*_1_ at week 6. Table 5 shows results of measuring the concentration of *GAG* in untreated and colchicine-treated cells.

The results of the study demonstrate that the *GAG* concentration in cells can be measured and quantified using an MRI. These MRI measurements show usefulness in healthy and cancerous breast cells. Additionally, we showed that the *GAG* concentration can be estimated when treated with colchicine cells.

## 4. Discussion

There is a clear need for technology to track cellular changes during treatment. As demonstrated by the recent development of cell therapy and potential applications of stem cells for organ regeneration, an MRI can provide advantages in this research. MRI employs the principles of quantum physics for the interrogation and depiction of biochemistry in situ. Analysis of the literature demonstrates that MRI is finding increasing application in biomedical fields. Recent advances generally focus on the quantification of metabolic profiles of cancer tissues in vitro, ex vivo, and in vivo, with an increasing likelihood for translation into clinical diagnostics. Tumors are unorganized organs that contain many different cell types. In recent years, many studies have reported that primary tumors contain fibroblasts/myofibroblasts (carcinoma-associated fibroblasts), mesenchymal cells such as pericytes/mural cells, and other vascular smooth muscle cells.

Colchicine retains all the properties discussed above but also provides the opportunity to attach an antibody or other ligand of interest to the colloid. As a result, new biological properties can be conferred to the reagent, such as targeting to a specific cell. The present study demonstrates that colchicine treatment in the 3D cultures of MCF-7 breast cancer cells reduced cell viability and was accompanied by measurable changes in the *GAG* concentration, as assessed by MRI. Importantly, HER-2-positive cells showed higher baseline *GAG* levels compared to HER-2-negative cells, and colchicine decreased the *GAG* content in both lines. These findings indicate a possible link between HER-2 signaling and extracellular matrix composition, and they highlight the usefulness of FCD-MRI as a non-invasive method for monitoring tumor-related changes. A concentration of 1000 nM (nanomolar) for colchicine is a very high concentration, often used in laboratory settings (in vitro) to study its effects on cells, but far higher than the therapeutic range seen in patients during clinical use. In clinical practice for treating or preventing gout attacks, plasma concentrations remain in the nanomolar to low micromolar range, with steady-state levels between 1.25 and 21 nM after a single oral dose, according to the Thieme Group and Mayo Clinic. Higher concentrations, such as 1000 nM, are used in laboratory experiments to investigate the drug’s mechanisms of action on cells. The significance of the 1000 nM concentration depends entirely on the experimental context. In a laboratory setting, it might be a standard test concentration; however, in a patient, this level is significantly higher than recommended and could be dangerous [72].

When compared with earlier reports, our observations emphasize cell-type-specific differences. Thomas et al. showed that colchicine reduced sulfate incorporation into *GAG*s in precapillary endothelial cells [73], while Jansen et al. described the inhibition of *GAG* synthesis and secretion in cartilage and chondrocytes after microtubule disruption [74]. In contrast, our results in breast cancer cells point to a reduction in the total *GAG* content detectable by MRI, which underlines the importance of considering tumor-specific responses to microtubule-targeting drugs.

This is mainly due to substantial methodological differences between our approach, which employs FCD-MRI, and classical biochemical techniques commonly used in previous studies. While spectrophotometric assays or [^35^S]-sulfate incorporation methods primarily quantify the synthesis or mass of selected *GAG* fractions, FCD-MRI reflects the fixed charge density within the extracellular matrix, which is strongly influenced by the degree of sulfation and water mobility. These differences may result in apparent discrepancies—for example, reduced sulfation without a change in the total *GAG* mass would lower the FCD-MRI signal but might remain undetected in mass-based assays. Furthermore, variations in cell models (endothelial cells, chondrocytes, and bone tissue vs. breast cancer cell lines), colchicine concentrations, and exposure times reported in the literature may also contribute to divergent observations.

Previous studies have also demonstrated the role of hyaluronan and other *GAG*s in breast cancer progression and metastasis [75,76,77,78,79]. The higher *GAG* levels observed in HER-2-positive MCF-7 cells in our study may be consistent with these reports, suggesting that receptor tyrosine kinase signaling influences *GAG* metabolism. Indeed, studies have shown that the activation of tyrosine receptors affects the expression of hyaluronan synthases and *GAG* sulfotransferases, leading to changes in the extracellular matrix composition [80,81]. Taken together, our data support the concept that HER-2 overexpression modifies *GAG* pathways, potentially affecting tumor progression and therapeutic response. MRI-based *GAG* quantification offers significant added value by providing a non-invasive, spatially resolved, and in vivo assessment of tissue composition, which is crucial for diagnosing early disease, monitoring treatment efficacy, and evaluating tissue engineering constructs, surpassing established biochemical assays that are often invasive, destructive, and provide only bulk measurements. Techniques like Chemical Exchange Saturation Transfer (gagCEST) or dGEMRIC (delayed Gadolinium-Enhanced Magnetic Resonance Imaging of Cartilage) can measure the *GAG* content without needing to take tissue samples.

MRI allows for the creation of detailed maps of *GAG* distribution, revealing regional variations in *GAG* content within a tissue, which is not possible with bulk biochemical assays. Limitations of *GAG* quantification for HPLC, NMR, or MS biochemical assays are as follows: biochemical methods require tissue biopsy, which is an invasive procedure; the process of performing a biochemical assay typically destroys the tissue sample, preventing further analysis; standard biochemical assays provide an average *GAG* concentration for the entire tissue sample, lacking the spatial resolution to identify localized changes; and the amount of *GAG* that can be measured is limited by the size of the tissue sample that can be obtained. The research focuses on HER-2’s role in cancer (especially breast and gastric cancer), its association with prognosis and treatment responses, and its impact on other signaling pathways like the cGAS-STING pathway or inflammation.

HER-2 (also known as ERBB2) regulates glycosaminoglycans (*GAG*s) by modulating the expression of specific proteoglycans, such as biglycan and syndecans. For example, HER-2/neu-mediated oncogenic transformation leads to the silencing of biglycan expression, which promotes tumor cell proliferation and migration. Conversely, the reconstitution of biglycan expression inhibits these processes. Furthermore, HER-2-positive breast cancer cells interact with heparan sulfate (HS) *GAG* chains on cell surface proteoglycans like syndecan and glypican, with the structure and function of these HS chains playing a role in regulating cell proliferation, motility, and metastasis [82].

In HER-2-positive cancers, the oncogenic signaling pathway suppresses biglycan expression. This reduction in biglycan, which acts as a tumor suppressor, supports the uncontrolled growth and spread of cancer cells [83]. Heparan sulfate (HS) chains, which decorate syndecan and glypican proteoglycans [84], are crucial for cancer cell behavior. HER-2-positive cells exhibit altered HS structures, which are linked to increased proliferation and metastasis by influencing cell–extracellular matrix (ECM) interactions and signaling pathways [83]. HER-2 influences the expression and function of *GAG*s by activating or inhibiting downstream signaling pathways. For instance, inhibition of the protein kinase C (PKC) pathway can restore biglycan expression in HER-2-transformed cells, highlighting an indirect mechanism of *GAG* regulation [85]. *GAG* quantification uses various methods, including dye-binding assays (like Dimethylmethylene blue and Safranin O), chromatographic techniques (HPLC, GC), and mass spectrometry (LC-MS/MS). Dye-binding methods are common but less specific, while chromatographic and mass spectrometry approaches offer greater sensitivity and allow for the identification of specific *GAG* types.

Dye-binding methods using Dimethylmethylene blue (DMMB) utilize a cationic dye that binds to sulfated *GAG*s, allowing for quantification by measuring the dye’s absorbency. It is widely used but can be affected by protein contamination and dye purity [86]. Similarly to DMMB, Safranin O is a cationic dye that binds to the anionic charges of *GAG*s, particularly chondroitin sulfate and keratan sulfate. Using Alcian Blue is a relatively fast method for *GAG* quantification, though it also struggles to distinguish between different *GAG*s [86]. A chemical reaction with carbazole can be used to quantify *GAG*s, with miniaturized versions offering increased sensitivity [85,86].

Chromatographic and Spectroscopic methods such as High-Pressure Liquid Chromatography (HPLC) are highly sensitive techniques that can quantify major *GAG* classes and even distinguish between *GAG*s with different sugar backbones (e.g., glucosamine vs. galactosamine *GAG*s) [85,86]. Mass spectrometry, especially liquid chromatography–tandem mass spectrometry (LC-MS/MS), provides high sensitivity, accuracy, and specificity, allowing for the precise measurement of *GAG*s and their specific structural subsets [86]. Gas Chromatography (GC) can be used for *GAG* detection, but it may have limited sensitivity and separation efficiency for these complex molecules [85,86].

An Enzyme-Linked Immunosorbent Assay (ELISA) can also be used for *GAG* detection. Histological Methods: Quantitative analysis of *GAG*s can also be performed on stained histological sections using microscopy and computer-based image analysis, such as with Safranin O-stained cartilage sections [85,86].

The methodological novelty of our study lies in the application of FCD-MRI. In contrast to classical biochemical approaches, which rely on destructive sampling and provide only endpoint measurements, MRI enables the repeated, non-invasive quantification of *GAG*s in the same culture over time. This approach is particularly valuable in 3D hollow fiber bioreactor systems, where the stable environment supports the long-term monitoring of treatment effects under near-physiological conditions.

Nevertheless, some limitations must be acknowledged. Our experiments were limited to a single colchicine concentration and relatively short exposure time. In addition, while FCD-MRI provides information on fixed charge density, it is strongly influenced by the sulfation degree and water mobility, which complicates direct comparison with mass-based biochemical assays [74,75]. Future research should therefore explore multiple drug concentrations, extended treatment periods, and complementary biochemical techniques to validate MRI-based measurements.

In summary, this study shows that colchicine reduces both the cell viability and *GAG* concentration in 3D cultures of MCF-7 breast cancer cells. HER-2-positive cells exhibited higher baseline *GAG* levels, suggesting a link between receptor signaling and extracellular matrix remodeling. The application of FCD-MRI provided novel, non-invasive insights into these processes, demonstrating its potential as a translational tool for monitoring the effects of anticancer treatment.

## 5. Conclusions

MRI is a powerful combination of magnetic resonance spectroscopy, a transformative tool in biochemistry, and is a crucial component of clinical care. Multinuclear MRSI paired with recent technological advances shows tremendous promise for improving our ability to characterize and monitor breast cancer and its response to therapy. The study showed that the use of the MRI technique allows for the non-invasive and reproducible monitoring of changes in glycosaminoglycan (*GAG*) levels in three-dimensional cultures of colchicine-treated breast cancer cells. A decrease in cell viability and accompanying changes in *GAG* content were observed, with the MCF-7(HER-2) line having higher levels of these compounds than the MCF-7(Neo-4) line. The results suggest the existence of a relationship between HER-2 expression and the *GAG* metabolism in breast cancer cells.

The approach presented here shows the potential of MRI as a valuable tool in studying the response of cancer cells to treatment, allowing for multiple and non-destructive measurements under 3D culture conditions. Limitations of the work are the small number of data and the lack of parallel biochemical analyses, so further studies should include a wider range of colchicine concentrations, different exposure times, and a comparison of MRI results with classical *GAG* determination methods. These emerging fields will attempt to understand how newly discovered drugs can impact human physiology and disease.

## Figures and Tables

**Table 1:** Viability of untreated cells.

Number of Weeks in Culture	Viability of Untreated HMEC	Viability of Untreated MCF-7 Her 2	Viability of Untreated MCF-7 Neo 4
1	99 ± 2	98 ± 2	100 ± 2
3	95 ± 3	94 ± 3	95 ± 4
6	95 ± 5	94 ± 4	94 ± 4

**Table 2:** Viability of treated cells.

Number of Weeks in Culture	Viability of Treated HMEC	Viability of Treated MCF-7 Her 2	Viability of Treated MCF-7 Neo 4
1	73 ± 2	78 ± 2	65 ± 1
3	75 ± 6	64 ± 7	55 ± 4
6	56 ± 10	49 ± 10	47 ± 6

**Table 3:** *T*_1_ MRI tissue parameters of HMEC, MCF-7 HER-2-positive, and MCF-7 (Neo-4) HER-2-negative breast cancer cell lines for both untreated and colchicine-treated cells. Values are the mean and ± SD.

Number of Weeks in Culture	*T*_1_ (ms) Untreated HMEC	*T*_1_ (ms) Treated HMEC	*T*_1_ (ms) Untreated MCF-7 Her 2	*T*_1_ (ms) Treated MCF-7 Her 2	*T*_1_ (ms) Untreated MCF-7 Neo 4	*T*_1_ (ms) Treated MCF-7 Neo 4
1	2876 ± 111	2278 ± 34	2613 ± 209	2114 ± 76	2573 ± 112	2023 ± 36
3	2230 ± 67	1876 ± 12	2430 ± 216	1987 ± 31	2320 ± 162	1972 ± 11
6	1542 ± 56	1454 ± 10	1712 ± 111	1654 ± 16	1654 ± 110	1354 ± 10

**Table 4:** *T*_2_ MRI tissue parameters of HMEC, MCF-7 HER-2-positive, and MCF-7 (Neo-4) HER-2-negative breast cancer cell lines for both untreated and colchicine-treated cells. Values are the mean and ± SD.

Number of Weeks in Culture	*T*_2_ (ms) Untreated HMEC	*T*_2_ (ms) Treated HMEC	*T*_2_ (ms) Untreated MCF-7 Her 2	*T*_2_ (ms) Treated MCF-7 Her 2	*T*_2_ (ms) Untreated MCF-7 Neo 4	*T*_2_ (ms) Treated MCF-7 Neo 4
1	156 ± 11	123 ± 10	148 ± 19	121 ± 11	121 ± 10	119 ± 09
3	134 ± 10	122 ± 10	114 ± 11	109 ± 10	109 ± 12	98 ± 23
6	109 ± 4	91 ± 11	113 ± 20	91 ± 11	98 ± 16	78 ± 17

**Table 5:** Results of measuring the concentration of *GAG* in untreated and colchicine-treated cells.

Number of Weeks in Culture	Untreated HMEC	Treated HMEC	Untreated MCF-7 Her 2	Treated MCF-7 Her 2	Untreated MCF-7 Neo 4	Treated MCF-7 Neo 4
6	7.3 ± 3	9.1 ± 4	3.3 ± 3	2.5 ± 2	12.6 ± 4	8.6 ± 4

## Data Availability

The raw data supporting the conclusions of this article will be made available by the authors on request.
